# Herpes Simplex Virus Colitis in a Patient with Newly Diagnosed Crohn's Disease

**DOI:** 10.1155/2018/7591709

**Published:** 2018-06-14

**Authors:** H. Jafri, D. R. Kalina, T. Aziz, P. E. Serrano, S. Haider

**Affiliations:** ^1^Department of Internal Medicine, McMaster University, Hamilton, ON, Canada; ^2^Department of Internal Medicine, Division of Infectious Diseases, McMaster University, Hamilton, ON, Canada; ^3^Department of Pathology and Molecular Medicine, McMaster University, Hamilton, ON, Canada; ^4^Department of Surgery, McMaster University, Hamilton, ON, Canada

## Abstract

Herpesvirus colitis is a known cause of morbidity and mortality amongst immunosuppressed individuals. We present a case of HSV colitis following a diagnosis of Crohn's Disease and methylprednisolone therapy. Diagnosis was confirmed by immunohistochemical staining and supported by polymerase chain reaction (PCR) of cutaneous vesicles. The patient recovered following three weeks of acyclovir.

## 1. Background

The management of inflammatory bowel disease (IBD) continues to evolve with the introduction of more toxic immunosuppressive therapies [1, 2]. Furthermore, paradigm shifts in management of IBD has resulted in earlier use of corticosteroids, thiopurine antimetabolites (6-MP, azathioprine), methotrexate, calcineurin inhibitors, and anti-TNF agents [2]. While these treatments are effective in managing IBD, they can result in severe acute microbial infections, loss of immunologic control of chronic viral infection (HIV and hepatitis B/C), reactivation of latent infections (HSV, EBV, and CMV), and malignancies resulting from oncogenic viruses (HPV and EBV) [3–5].

Herpesvirus infections are common sequelae in the clinical course of patients being managed for IBD [2–5]. Although cytomegalovirus (CMV) is a common cause of tissue-invasive disease in these patients [1], herpex simplex virus (HSV) complicating the course of an IBD flare is a rare phenomenon. There have been only 4 published cases of HSV superinfection in the background of IBD [6–9].

## 2. Case Report

A 61-year-old female presented to hospital with a 2-week history of profound diarrhea and vomiting. The patient also complained of dull abdominal pain that temporarily resolved with bowel movements. She denied fevers, weight loss, exposure to sick contacts, external food sources, and a travel history. There were no extraintestinal manifestations of inflammatory bowel disease (IBD), such as arthralgias, uveitis, episcleritis, oral ulcers, and aphthous ulcers.

She was admitted with an initial diagnosis of viral gastroenteritis and treated with supportive therapy. Stool testing for *Clostridium difficile*, ova and parasites, viral PCR, and bacterial cultures were negative. A CT abdomen revealed diffuse edematous changes in the ascending colon, transverse colon, and descending colon, as well as hyperemia in the mesentery—indicating colitis.

On her second day of admission, the patient developed bloody diarrhea, prompting a colonoscopy by the gastroenterology service. The colonoscopy revealed severe inflammation with large (0.5–3 cm) deep punched-out ulcers, spontaneous bleeding, bridging mucosa, and patchy erythema affecting 80–90% of the mucosa from the cecum to the transverse colon, with rectal sparing. Several scattered aphthous ulcers were also noted.

Multiple biopsy samples were taken from the colon—they revealed severe chronic colitis with focal areas of ulceration, focal cryptitis, and architectural distortion. Esophagogastroduodenoscopy (EGD) was normal. The results of the colonoscopy were consistent with Crohn's disease, and the patient was treated with intravenous methylprednisolone 80 mg for seven days.

On day 3 of admission, the patient developed fevers, chills, significant right-sided parotid swelling, erythema, and tenderness. Ultrasound of the right neck revealed parotitis with no abscess. Blood cultures revealed MSSA bacteremia, with parotitis being the presumed source. Transesophogeal echocardiogram was negative, and the patient was subsequently treated with supportive therapy and fourteen days of cefazolin.

The patient had a stable clinical course for the next thirteen days. However, on postadmission day 17, the patient developed severe abdominal pain and hemodynamic instability. On examination, the patient's abdomen was diffusely tender and rigid, suggesting peritonitis. Abdominal X-ray revealed features of colitis complicated by perforation. CT abdomen revealed bowel wall edema, pneumatosis, and focal perforation involving the anterior wall cecum/ascending colon, with extensive pneumoperitoneum. The patient was initially resuscitated and taken to the operating room the next day for a laparotomy. Findings in the operating room revealed microperforations of the cecum, large perforation at the splenic flexure, and fecal peritonitis. A subtotal colectomy was performed, as well as intra-abdominal and subphrenic abscesses were drained. The patient remained intubated postoperatively and was transferred to the intensive care unit (ICU). Concurrent blood cultures revealed *Enterobacter cloacae* bacteremia from an intra-abdominal source, and the patient was treated with intravenous ciprofloxacin. However, the patient remained intubated and required continued use of vasopressors in the ICU. Upon examination, the surgical wound demonstrated signs of infection, which along with the patient's clinical status suggested persistent intra-abdominal sepsis. On hospital day 25, the patient went to the operating room again for a washout of the abdomen and creation of a rectal stump mucous fistula. Tissue cultures revealed growth of *Escherichia coli* and *Enterococcus faecium*. Antimicrobical treatment with intravenous meropenem and intravenous metronidazole was commenced, for broad coverage of intra-abdominal abscess and sepsis.

**Figure 1:**
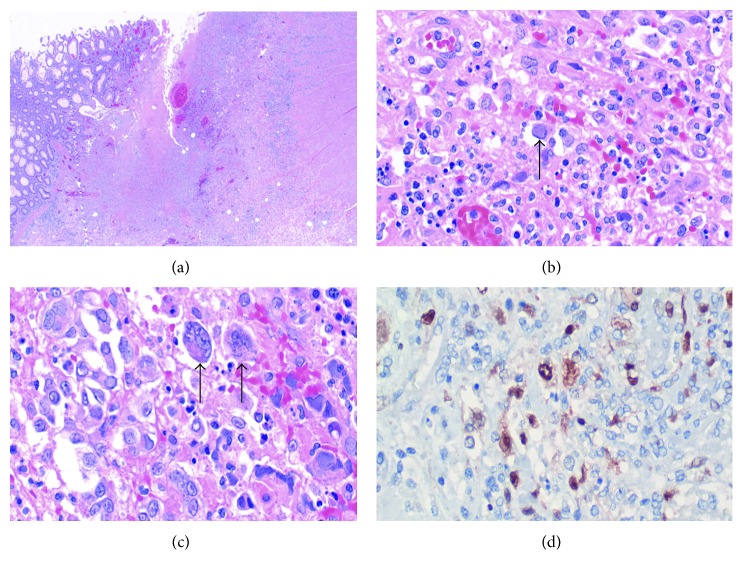
(a) Section from the ulcerated colonic mucosa with deep fissuring ulcer. Viral cytopathic changes are noted. (b, c) Giant cells within ulcer with characteristic intranuclear Cowdry type A inclusions of HSV-1 and HSV-2. Black arrows point to individual giant cells with Cowdry type A inclusions. (d) Immunohistochemistry study for HSV-2 antibody highlighted by the brown nuclear staining.

The surgical biopsy from subtotal colectomy revealed extensive diffuse mucosal inflammation, acute ulceration, and mucosal and transmural ischemic changes from the ileum to the distal colon (Figure 1(a)). The ulcerated area exhibited granulation tissue that contained cells that diffusely exhibited intranuclear inclusions, morphologically and immunohistochemically consistent with herpes simplex virus (HSV-1 and HSV-2) (Figures 1(b)–1(d)). Immunohistochemistry for CMV was negative. The patient was started on treatment with intravenous acyclovir.

Upon physical examination, the patient had developed lesions on the tongue, with visible dry blood. No lesions were seen on the lips. Furthermore, the patient had vesicular eruptions on the lower abdomen and inner thigh. The fluid from the tongue and abdominal lesions was also positive for HSV-2 DNA on a PCR-based assay, thus suggesting a disseminated HSV-2 infection. Viral PCR was negative for HSV-1 and varicella-zoster. The patient received treatment with intravenous acyclovir 10 mg/kg for three weeks.

The patient had a prolonged stay in the ICU spanning 42 days. She continued to make a recovery on the ward, as her hospital stay was further complicated by catheter- and line-related infections, as well as poor nutrition and muscle atrophy. The patient was discharged from the hospital on day 122.

## 3. Discussion

The evolution of immunomodulatory therapies for the management of IBD has improved prognosis in this patient population, with the caveat of increased risk of infection with various microorganisms [2–5]. CMV colitis is a common complication in the clinical course of IBD—among IBD patients with acute colitis, the prevalence of CMV superinfection is 21–34% [1]. In contrast, HSV superinfection in the context of IBD has been rarely described in the literature [6, 10]. Along with the use of immunosuppressive agents, persistent inflammation is also a risk factor for developing HSV superinfection [4].

While it is important to consider the possibility that HSV colitis was in fact a triggering condition rather than merely a superinfection, we consider this to be less likely in our presented case. Colonoscopic biopsies that had been taken two weeks prior to those showing evidence of HSV colitis showed evidence of colitis suggestive of inflammatory bowel disease in the absence of signs of HSV colitis. Given the relatively low specificity of serologic tests for HSV [11], especially in active infection [10], it is difficult to speculate if there had been increasing burden of viral disease leading to the development of the patient's IBD.

It is essential to recognize HSV colitis as a clinical entity in this patient population. A higher index of suspicion will lead to earlier diagnosis and management, which can significantly alter the clinical course and ultimately improve patient outcomes [12]. In such cases, the physical examination can be helpful as it was in our case—there was evidence of vesicular lesions indicating mucocutaneous manifestation of active HSV infection [7]. However, the most essential component in the diagnosis of HSV colitis is immunohistochemistry. The diagnosis can only be confirmed with adequate tissue samples sent for immunohistochemical analysis [7, 8, 13]. Similar to CMV colitis, HSV colitis can present with intranuclear eosinophilic inclusion bodies [7, 8, 13]. However, immunohistochemistry for CMV was negative in the colonic sample in our case, while it was positive for HSV. It is essential to appropriately test for all potential culprit microorganisms; otherwise, the diagnosis can be missed. Although CMV is the more common culprit, HSV must be ruled out immunohistochemically, especially if testing for CMV is negative.

Although early diagnosis and treatment is the current clinical standard of care, future strategies may involve prophylactic treatments in patients receiving immunosuppressive therapies for IBD [5]. Prophylactic antiviral therapy may have a role in immunosuppressed patients with serological results consistent with latent viral infections or a history of complicated viral infections.
